# An open-label continuation trial of sirolimus for tocilizumab-refractory idiopathic multicentric Castleman disease: Study protocol for an investigator-initiated, multicenter, open-label trial (SPIRIT compliant)

**DOI:** 10.1097/MD.0000000000023291

**Published:** 2020-12-11

**Authors:** Tomohiro Koga, Sachiko Takemori, Naoko Hagimori, Shimpei Morimoto, Remi Sumiyoshi, Toshimasa Shimizu, Naoki Hosogaya, Chizu Fukushima, Hiroshi Yamamoto, Atsushi Kawakami

**Affiliations:** a Division of Advanced Preventive Medical Sciences, Department of Immunology and Rheumatology; b Center for Bioinformatics and Molecular Medicine, Nagasaki University Graduate School of Biomedical Sciences; c Nagasaki University Hospital, Clinical Research Center, Nagasaki; d Translational Research Center for Medical Innovation, Foundation for Biomedical Research and Innovation at Kobe, Kobe, Japan.

**Keywords:** idiopathic multicentric Castleman disease, mammalian target of rapamycin, open-label, sirolimus, tocilizumab-resistant

## Abstract

**Background:**

Interleukin 6 (IL-6) inhibitors are the first-line treatment for idiopathic multicentric Castleman disease (iMCD); however, there is no established treatment for cases that are resistant to IL-6 inhibitors. Although sirolimus, a mammalian target of rapamycin inhibitor, has been suggested to be effective in patients with iMCD, the long-term safety and efficacy of sirolimus on individuals with IL-6 inhibitor-resistant iMCD have not been evaluated.

**Methods/Design:**

In this investigator-initiated, multicenter, open-label trial, the long-term safety of sirolimus will be evaluated in patients participating in a placebo-controlled, randomized, double-blind, parallel-group trial on tocilizumab (TCZ)-resistant iMCD. The study will be conducted in 7 centers in Japan. This trial will be promptly started after the evaluation and examination for 16 weeks in the preceding study. The trial will be completed by the time the drug is approved for iMCD treatment in Japan. The primary endpoint is the incidence of adverse events. The secondary endpoints include the following: the levels of hemoglobin, albumin, and C-reactive protein; change in CHAP score; physician global assessment (100-mm visual analog scale); patient global assessment (100-mm visual analog scale); and lymph node changes in subjects with lymphadenopathy.

**Discussion:**

This clinical trial will provide evidence regarding the long-term safety of sirolimus as a potential novel therapeutic agent for patients with tocilizumab-resistant iMCD.

**Trial Registration number:**

jRCT2051200050

## 1 Introduction

Castleman disease (CD) is a lymphoproliferative disease first described by Benjamin Castleman in 1956.^[1]^ CD was subsequently classified into unicentric CD, which is a localized form with lymph node involvement, and multicentric CD (MCD), which is a multicentric form of the disease.^[2]^ Lymphadenopathy in patients with unicentric CD is localized and is generally asymptomatic and does not show abnormalities on clinical examination. Conversely, MCD is a systemic disease associated with multiple lymphadenopathies and symptoms such as fever, night sweats, weight loss, and body malaise.^[3–5]^

MCD can be divided into human herpes virus-8 infection-associated MCD and idiopathic MCD (iMCD)^[2]^; however, iMCD can cause organ damage and secondary amyloidosis without appropriate treatment and can be a major cause of death. It reduces the quality of life and shortens life expectancy. Interleukin 6 (IL-6) is a significant cytokine in the MCD pathogenesis,^[6]^ although the mechanisms underlying the IL-6 overproduction in MCD patients are not fully understood.

The presence of patients with iMCD who do not respond to anti-IL-6 therapy suggests that pro-inflammatory cytokines other than IL-6 also play a significant role in the iMCD pathogenesis. A report on the efficacy of sirolimus, a mammalian target of rapamycin (mTOR) inhibitor, in refractory iMCD^[7]^ suggests the phosphatidylinositol-3-kinase (PI3K)/Akt/mTOR pathway activation in the iMCD pathogenesis. The inhibition of this pathway, in addition to inhibiting the T and B cell proliferation that are activated in iMCD, has a suppressive effect on vascular endothelial growth factor, which may be particularly effective in some IL-6-independent iMCD.

We are currently recruiting patients with iMCD who completed a phase III, investigator-initiated, multicenter, double-blind, randomized, parallel-group trial to confirm the long-term safety and efficacy of sirolimus on individuals with tocilizumab (TCZ)-resistant iMCD.^[8]^ Herein, we describe the final protocol (version 2.0; June 26, 2020) for this study. The results of this study are expected to provide evidence regarding the long-term safety and efficacy of sirolimus for the treatment of TCZ-resistant iMCD patients.

## 2 Methods/design

### 2.1 Study design

The present study has been designed in accordance with the Standard Protocol Items: Recommendations for Interventional Trials and Consolidated Standards of Reporting Trials 2010 guidelines.^[9,10]^ This is an open-label investigator-initiated, multicenter study of the long-term safety and efficacy of sirolimus in patients with TCZ-resistant iMCD.

This study will be conducted at 7 centers in Japan and will be performed in accordance with the principles of the Declaration of Helsinki^[11]^ and the Japan Good Clinical Practice. The study was registered to the Japan Registry of Clinical Trials as jRCT2051200050 and was approved by the Institutional Review Board of the Daini Osaka Police Hospital.

### 2.2 Participant recruitment

Participants will be recruited at the Nagasaki University Hospital, Jikei University Hospital, Kanazawa Medical University Hospital, Kyoto University Hospital, Sumitomo Hospital, Daini Osaka Police Hospital, and Kyushu University Hospital. Based on the inclusion and exclusion criteria, all eligible participants will be selected and approached based on information from the electronic health record from these 7 hospitals. Participants will be provided with an explanation regarding the study by their treating pediatrician/rheumatologist and clinical research coordinator and will be asked to voluntarily sign an informed consent form before their participation.

### 2.3 Inclusion criteria

Patients who meet the following inclusion criteria are eligible for this study:

1) patients who completed the 16-week treatment with an investigational drug in the preceding trial (jRCT2071190029); and
2) patients who received a thorough explanation of the contents of explanatory documents and other matters concerning clinical trials, understood the contents thereof, and provided written consent based on their free will to participate in this trial.

### 2.4 Exclusion criteria

Patients with any of the following at the time of screening will be excluded:

1) patients with an Eastern Cooperative Oncology Group Performance Status of 4;
2) patients who cannot use appropriate contraception during the study drug administration period or 12 weeks after the last treatment with the study drug;
3) female patients during lactation or pregnancy;
4) patients with complications of serious diseases that are deemed unsuitable for the clinical trial by the investigator or sub-investigator;
5) patients whose condition in the preceding trial is deemed unsuitable for continued treatment by the investigator or sub-investigator; and
6) other patients deemed inappropriate by the investigator or sub-investigator.

### 2.5 Study protocol

The study is a continuation study in patients who had completed the 16-week treatment period of the investigational drug in the previous study. The investigator or sub-investigator then considers whether the patient is eligible for the study, taking into consideration the patient's health status, severity, and symptoms of the primary disease and complications, age, and ability to consent. The investigator or sub-investigator explains the consent to the subjects who are expected to complete the preceding trial and who have completed the 12-week observation and examination of the preceding study.

If the observation and examination at Week 0 is conducted on the same day as the Week 16 of the preceding study, written consent should be obtained from the subjects and others before the initial observation and examination at Week 16 of the preceding study. If the study does not commence on the same day as the 16-week follow-up study, written consent should be obtained from the subject or others within 28 days of the completion of the 16-week follow-up study.

**Figure 1:**
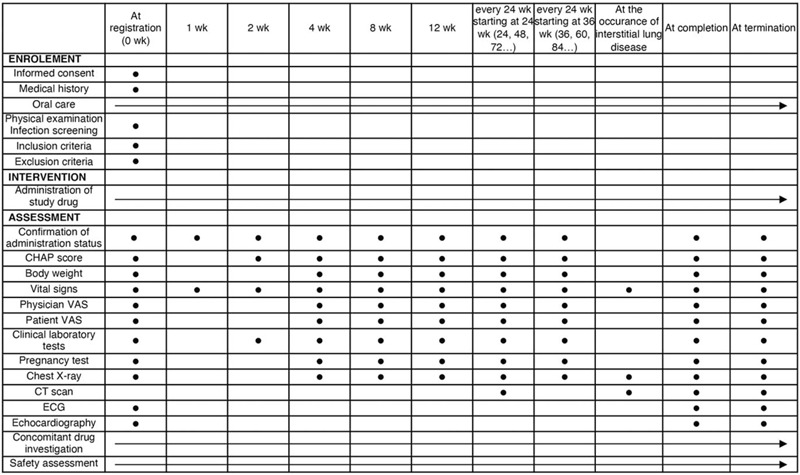
Treatment schedule and outcome measures. CT = computed tomography, ECG = electrocardiogram, VAS = visual analog scale.

The investigator or sub-investigator conducts the observation and examination at week 0 of the trial to determine the eligibility of the subjects based on the selection and exclusion criteria. The overlapping items of the observation and examination at Week 0 of this study with those of the preceding 16 weeks can be substituted. All patients who meet the inclusion criteria and do not meet any of the exclusion criteria will receive the investigational drug at 2 mg orally once daily. Therefore, all participants who were assigned to the placebo group in the preceding study will also receive the investigational drug. In principle, the first dose of the investigational drug is administered on the same day as the eligibility verification completion date. If the discontinuation criteria are met, the medication is discontinued, and the prescribed tests are performed. After the visit date (i.e., the initial investigation day of the investigational drug), the investigators will continue to administer the investigational drug and conduct necessary examinations and surveys in accordance with the schedule presented in Figure 1.

The planned clinical trial period is from the date of approval by the head of the investigational site to the date of obtaining a manufacturing and marketing approval for iMCD. The clinical trial is terminated when it is decided to discontinue development for this indication.

### 2.6 Adverse events

If an adverse event is observed in a patient during the study period, the investigator of each medical institution should immediately take appropriate measures and record that event in the clinical records and case reports. The severity of adverse events should be determined according to the Common Terminology Criteria for Adverse Events, version 5.0. A serious adverse event is defined as any untoward medical event that occurs at any dose, results in death, is life-threatening, requires inpatient hospitalization, or prolongation of existing hospitalization, results in persistent or significant disability or incapacity, or causes a congenital anomaly or birth defect.

The study design includes an independent efficacy and safety assessment committee that will review the ongoing safety data in an unblinded manner in accordance with the Standard Operating Procedures for Clinical Trials, Japan Medical Association (http://www.jmacct.med.or.jp/).

The investigational drug may increase the risk of developing interstitial lung disease (ILD). If ILD is suspected or developed during the study drug administration period, the investigator or sub-investigator should examine the patient (blood pressure, pulse rate, temperature, respiratory rate, percussion, and auscultation, SpO2) and perform chest X-ray, CT scan, and clinical examination to confirm the presence of ILD. If the patient has ILD, its symptoms and severity should be confirmed, and the drug should be withdrawn or discontinued according to the guidelines, depending on the symptoms and severity of the ILD. The investigator or sub-investigator should send the chest X-ray and CT scan image data to the efficacy and safety assessment committee and follow the standard operating procedures of the committee.

### 2.7 Outcome

The primary endpoint of this study is the incidence of adverse events. The secondary endpoints are efficacy and safety categories.

#### 2.7.1 Efficacy

We will evaluate the efficacy of the investigational drug based on the following parameters:

1) hemoglobin (g/dL): change from the baseline at 2, 4, 8, 12, and 24 weeks, every 12 weeks thereafter, and at the time of the study completion or drug discontinuation;
2) albumin (g/dL): change from the baseline at 2, 4, 8, 12, and 24 weeks, every 12 weeks thereafter, and at the time of the study completion or drug discontinuation;
3) C reactive protein (mg/dL): change from the baseline at 2, 4, 8, 12, and 24 weeks, every 12 weeks thereafter, and at the time of the study completion or drug discontinuation;
4) Physician Global Assessment (disease activity assessment, 100-mm visual analog scale): change from the baseline at 4, 8, 12, and 24 weeks, every 12 weeks thereafter, and at the time of the study completion or drug discontinuation;
5) Patient Global Assessment (disease activity assessment, 100-mm visual analog scale): change from the baseline at 4, 8, 12, and 24 weeks, every 12 weeks thereafter, and at the time of the study completion or drug discontinuation;
6) lymph node changes in subjects with lymphadenopathy: change from the baseline at 24 weeks, every 24 weeks thereafter, and at the time of the study completion or drug discontinuation as well as changes in the number of lymph nodes >10 mm in diameter from the baseline at 24 weeks, every 24 weeks thereafter, and at the time of the study completion or drug discontinuation;
7) CHAP score: change from the baseline at 2, 4, 8, 12, and 24 weeks, every 12 weeks thereafter, and at the time of the study completion or drug discontinuation; and
8) CHAP score minus C reactive protein score: change from the baseline at 2, 4, 8, 12, and 24 weeks, every 12 weeks thereafter, and at the time of the study completion or drug discontinuation.

#### 2.7.2 Safety

The safety evaluation indices of this clinical trial are as follows:

1) adverse events, including the incidence rates of adverse events, serious adverse events, and side effects;
2) laboratory tests, including hematological examination, blood biochemical examination, and urinalysis; and
3) all medically significant indicators, including physical findings, vital signs, imaging tests, electrocardiogram results, echocardiographic findings, etc.

### 2.8 Data collection and management

The investigators will be provided access to an online, web-based, electronic data capture system. Only the investigator will be able to enter and modify data in the electronic case report form. All study findings and documents will be regarded as confidential. Patients will be identified on the electronic case report form by their patient number, but not by name. Document confidentiality must be maintained by the investigator to ensure the anonymity of the participants.

During the study, a sponsor-investigator will perform regular site visits to review protocol compliance, conduct source data verification, assess drug accountability and management, assess laboratory procedures, and ensure that the study is being conducted according to pertinent regulatory and protocol requirements. The sponsor-investigator controls the trial quality in accordance with the protocol, each standard operating procedure, and the trial monitoring/audit protocol. The primary operations are as follows:

1) hold and explain the protocol to the investigator or trial collaborators upon trial initiation or as necessary to ensure an accurate protocol understanding and judgment and evaluation standardization;
2) subject eligibility will be automatically checked by an electronic data capture system to eliminate selection and exclusion violations;
3) the monitor should visit the institution on a regular basis to ensure that the protocol and good clinical practices are followed to ensure proper conduct of the trial and to confirm data accuracy;
4) records and reports concerning the operation of clinical trials, data collection, data management, statistical analysis, and adverse event analysis shall be performed in accordance with standard operating procedures, and inspections and confirmations shall be made;
5) to ensure that the clinical trial is properly conducted, the sponsor-investigator will confirm the quality of each development activity through quality control. Auditors independent from departments involved in the drug development and implementation will systematically investigate and confirm clinical trial operations and documents in accordance with standard operating procedures for the implementation of audits; and
6) all source documents will be made available for review at the request of the sponsor-investigator or national or international regulatory authority.

### 2.9 Statistical analysis

The details of the statistical analysis will be documented in the statistical analysis plan. The statistical analysis plan will be signed off prior to the unblinding of the preceding double-blind study.^[8]^ Safety analysis set (SAS) will consist of all subjects who will have received at least 1 dose of the study drug in the present study. Full analysis set (FAS) will consist of the SAS subset who will have been administrated the study drug at least once in the present study and who will have result measuring at least one of the efficacy endpoints after the first administration of the investigational drug. Modified FAS (mFAS) will consist of the FAS subset that will have never changed the TCZ dose amount from the randomization in the preceding double-blind study^[8]^ to the end of the observation of the subject. Per-protocol set (PPS) will consist of the FAS subset excluding those with major deviations from the protocol of the present study. In the PPS analyses, the subjects who will have received the treatment of the opposite treatment group, against the allocation at the randomization in the preceding double-blind study,^[8]^ will be included in the opposite treatment group against the allocation at the randomization. We will perform statistical analyses for the primary endpoint using SAS and for the secondary endpoints using FAS, modified FAS, and PPS.

As a primary analysis, safety evaluation summaries will be presented as cross-tables of adverse events by the treatment group, causality and severity will be created; in a secondary analysis, the period of the adverse event onset will be included in the cross-tabulation factors. The event names will be defined by the system organ classes and preferred terms in the Medical Dictionary for Regulatory Activities terminology. The time-series fluctuation of the numerical measuring results from each patient will be plotted for each safety endpoint. Moreover, we will calculate summary statistics for each endpoint at each time point.

As for the measuring results of the outcomes listed in the section 2.7, variation through the observation period will be estimated by a mixed-effect model with random intercepts of subjects.

## 3 Discussion

Treatment with sirolimus achieved clinical remission in IL-6 inhibitor-resistant iMCD cases, suggesting an increased PI3K/Akt/mTOR pathway activity in iMCD patients^[7,12,13]^. To confirm these observations, we designed the “a randomized, double-blind, placebo-controlled, parallel-group trial of sirolimus for TCZ-resistant idiopathic multicentric CD” as a preceding study.^[8]^ This open-label extension study aimed to evaluate the long-term safety and efficacy of sirolimus in patients who completed the preceding study.

Sirolimus has already been approved for lymphangioleiomyomatosis (LAM) treatment in Japan in 2014, and the long-term safety profile of sirolimus has been previously shown.^[14]^ In addition, sirolimus has been approved by the Food and Drug Administration to prevent acute rejection in kidney transplantation for 20 years, and the evidences on its long-term safety are accumulating.^[15,16]^ These data demonstrate the long-term safety of using sirolimus in LAM and post-renal transplant patients in the real-world clinical practice. However, iMCD patients are more likely to receive concomitant immunosuppressive agents, such as TCZ and glucocorticoids, than patients with LAM. Furthermore, sirolimus is susceptible to CYP3A4 inhibitors and inducers, and the different concomitant medications used may result in different toxicities compared with LAM. In this study, we will determine whether the effects of sirolimus are attenuated by long-term administration, in addition to its tolerability to the long-term administration.

This trial will evaluate the long-term safety and efficacy of sirolimus in patients with TCZ-resistant iMCD. It will contribute to the identification of the effects of long-term sirolimus use in organ damage associated with chronic inflammation. These findings may support the evidence of mTOR-targeted therapy in patients with severe iMCD.

## Acknowledgments

The authors would like to thank their colleagues and staff at the Rheumatology Department of Nagasaki University Hospital for their support.

## Author contributions

TK, NH, HY, and AK are responsible for conceiving and designing the trial, planning data analysis, drafting the manuscript, making the final decision to terminate the trial, and approving the final manuscript. TK, NH, ST, RS, TS, and CF will participate in data collection and are in charge of recruitment and treatment of patients. TK, NH, and SM are responsible for planning data analysis and analyzing the data resulting from the trial. All authors will have access to the interim results as well as the capacity to discuss, revise, and approve the final manuscript.

**Conceptualization:** Tomohiro Koga, Naoki Hosogaya, Hiroshi Yamamoto, Atsushi Kawakami.

**Formal analysis:** Shimpei Morimoto.

**Funding acquisition:** Atsushi Kawakami.

**Investigation:** Sachiko Takemori, Remi Sumiyoshi.

**Methodology:** Sachiko Takemori, Naoko Hagimori, Shimpei Morimoto, Toshimasa Shimizu, Naoki Hosogaya, Chizu Fukushima.

**Supervision:** Hiroshi Yamamoto, Atsushi Kawakami.

**Writing – original draft:** Tomohiro Koga.

**Writing – review & editing:** Tomohiro Koga, Sachiko Takemori, Naoko Hagimori, Shimpei Morimoto, Remi Sumiyoshi, Toshimasa Shimizu, Naoki Hosogaya, Chizu Fukushima, Hiroshi Yamamoto, Atsushi Kawakami.
